# Bridging the Gap Between Academic Research and Pragmatic Needs in Usability: A Hybrid Approach to Usability Evaluation of Health Care Information Systems

**DOI:** 10.2196/10721

**Published:** 2018-11-28

**Authors:** Devin M Mann, Sara Kuppin Chokshi, Andre Kushniruk

**Affiliations:** 1 Department of Population Health School of Medicine New York University New York, NY United States; 2 Medical Center Information Technology NYU Langone Health New York, NY United States; 3 School of Health Information Science University of Victoria Victoria, BC Canada

**Keywords:** software design, user-computer interface, medical informatics

## Abstract

**Background:**

Technology is increasingly embedded into the full spectrum of health care. This movement has benefited from the application of software development practices such as usability testing and agile development processes. These practices are frequently applied in both commercial or operational and academic settings. However, the relative importance placed on rapid iteration, validity, reproducibility, generalizability, and efficiency differs between the 2 settings and the needs and objectives of academic versus pragmatic usability evaluations.

**Objective:**

This paper explores how usability evaluation typically varies on key dimensions in pragmatic versus academic settings that impact the rapidity, validity, and reproducibility of findings and proposes a hybrid approach aimed at satisfying both pragmatic and academic objectives.

**Methods:**

We outline the characteristics of pragmatic versus academically oriented usability testing in health care, describe the tensions and gaps resulting from differing contexts and goals, and present a model of this hybrid process along with 2 case studies of digital development projects in which we demonstrate this integrated approach to usability evaluation.

**Results:**

The case studies presented illustrate design choices characteristic of our hybrid approach to usability evaluation.

**Conclusions:**

Designed to leverage the strengths of both pragmatically and academically focused usability studies, a hybrid approach allows new development projects to efficiently iterate and optimize from usability data as well as preserves the ability of these projects to produce deeper insights via thorough qualitative analysis to inform further tool development and usability research by way of academically focused dissemination.

## Introduction

### Background

Technological solutions are a dominant modality for improving health care delivery and are increasingly embedded into the full spectrum of health care workflows—patient, provider, system, and population. The growing integration of technology into health care has benefited from the application of software development practices such as agile development, user-centered design, human-computer interaction, and usability testing [1-4]. Usability testing has emerged as an important methodology in health informatics [5-8]. Although it can take various forms, usability testing refers generally to the evaluation of a digital tool involving the observation of end users as they interact with that tool to carry out representative tasks [9,10]; for example, a clinician (representative user) may be observed while interacting with a clinical decision support (CDS) module in the electronic health record (EHR) system [9,11]. Observations are recorded and analyzed for the purposes of gathering feedback for user-centered tool development.

Observations made during the testing and the recorded user interactions (typically captured using screen-recording software) are analyzed to varying degrees of depth to identify specific usability issues, such as problems with navigation or “pain points” with regard to tool compatibility with user workflow [9,12]. These practices are applied in both commercial or operational and academic settings; however, the relative importance placed on rapid iteration, validity, reproducibility, generalizability, and efficiency differs between the 2 settings, as do the needs and objectives of academic versus pragmatic usability evaluations [6,7].

### Serving the Needs of Academic Usability Evaluation

With interest increasing in conducting and reporting data from usability studies from an academic perspective, the relevant literature has seen a growing number of publications proposing best practices and minimum standards of rigor for usability research [5,13-17]. Statement on Reporting of Evaluation Studies in Health Informatics principles, for example, provide proposed guidelines for conducting and reporting evaluation studies, including explicit consideration of scientific background, study context, detailing of methods, results, and the discussion of implications and limitations [14,18-20]. Peute and colleagues have extended these ideas to the creation of guidelines for usability evaluations for academic reporting, adding descriptive data on study participants and discussion on the generalizability and reproducibility of the study [15].

These guidelines and practices can be seen as supporting a move toward a culture of “evidence-based” human factors work in health care, as described by Marcilly and other authors [5,13,15,17]. Many of these practices, such as including a minimum number of representative users that would allow for statistical analyses and conducting objective and replicable analyses of the resulting data, are documented in the academic literature [15]. However, despite these established practices, software development projects in real clinical contexts continue to routinely minimize the role of truly rigorous evaluation [15,18,21].

### Agile Development and Pragmatic Usability Evaluation

Although academically oriented usability studies value validity, reproducibility, and generalizability, those usability studies conducted in primarily pragmatic settings (eg, commercial or clinical settings) prioritize speed, efficiency, and the ability to inform rapid, agile development cycles [22]. Agile development refers to a set of software development practices that, in contrast to more linear and traditional “waterfall” approaches, value rapid, flexible, and iterative processes that heavily incorporate end user feedback [23,24]. Agile and user-centered techniques are increasingly written about in relation to person-centered health information technology (HIT) design [3,24-27]. Although the increased attention paid to usability research is indicative of its potential value, details on how to conduct usability research in a way that is agile and iterative while aligned with the goals and demands of academic research remain sparse [28]. This gap in knowledge as to how to balance or reconcile objectives in academic and pragmatic usability engineering in health care represents an important knowledge translation problem, which may be at the root of a number of issues regarding the lack of usability of systems and lack of end user adoption of many HIT systems [2,29-32].

### Academic Versus Pragmatic Usability: A Comparison of Features

Academic and pragmatic usability studies may employ similar methods but as described above, can be characterized by several key differentiating features reflecting differing priorities [12]. The differences in priorities reflect differences in both the goals of each type of project as well as the funding source of academic (typically grants) versus pragmatic usability studies. Importantly, these differences can create tension within teams seeking to meet both academic and pragmatic research and development goals, including many teams at academic health centers with a mandate to produce effective and timely production systems for real-world use in clinical contexts [2,12,20].

Table 1 compares and contrasts features of more rigorous academic usability with those of a purely pragmatic usability approach. As highlighted above, there are shortcomings to using each of these approaches alone; purely pragmatic projects tend to sacrifice the potential for producing evidence useful to the wider HIT community, whereas purely academic usability evaluation may produce some interesting findings but risk long, costly timelines that are incompatible with the pace of digital innovation today. Although the table illustrates essential differences and potential tensions between the 2 perspectives, it is important to acknowledge that in reality, usability evaluations vary widely and differences in features between academic and pragmatic approaches may not be clear-cut. The priorities listed for each approach can help research and development teams understand the trade-offs involved when making these decisions regarding usability evaluation design.

**Table 1:** Comparison of features of academic versus pragmatic usability testing.

Feature	Academic usability	Pragmatic usability
Objectives	Production of evidence regarding adaptation and development of tool types (eg, clinical decision support) and workflows for academic publication and dissemination Priority: rigor and reproducibility	Rapid iterative design and testing cycles to provide user feedback to product owners and developers Priority: speed and cost-effectiveness
Methodological approach	Direct observation Think-aloud Near-live Live testing	Direct observation Think-aloud Near-live Live testing using low-cost approaches
Setting	Variable (laboratory to *in situ*) Priority: high-fidelity, representative testing environment and tasks	Variable (laboratory to *in situ*) Priority: convenience over fidelity
Number of participants	10-15 participants (representative of end users) per user group for usability testing (potentially more if conducting statistical analyses) Priority: representativeness of user	<10 participants (typically minimum=4) Priority: convenience and managing time constraints
Data capture	Note taking Audio recordings Video recording Screen capture Data captured and transcribed for detailed analyses	Observational note taking Notes on debriefing interviews Real-time analysis of user-screen interaction
Termination criteria	Termination with data saturation for current iteration	Termination based on consensus, cost, and time constraints
Data analysis	Detailed qualitative analyses (including interrater reliability) of data captured: usability testing transcripts, screen captures, etc Quantitative analyses (eg, error rates, System Usability Scale scores, measures of clicking, eye tracking, etc)	Concise, structured summaries of findings based on notes from usability sessions and debriefings and notes from anecdotal and stakeholder feedback
Output	Detailed data tables and results reporting	Simple summary or table of problems and solutions
Dissemination	Publication of findings in peer-reviewed journals Priority: generalizability of results and scientific value	Final summary report presented to developers and management Priority: local (vs wider) distribution of findings for use to improve a specific system or interface
Time frame	Varies from weeks to months	Feedback from testing immediately or within days of testing

### Methodological Approaches, Setting, and Number of Participants

Although differing in objectives, data collection may be similar across the 2 approaches, including direct observation, the think-aloud method (users are asked to provide real-time, out-loud feedback while carrying out representative tasks), and near-live (observed use of the tool in a clinical simulation in realistic settings) and live usability testing (observed use of the tool postdeployment to discern outstanding issues with design or integration with workflows before wider implementation) [33,34]. The tools and methods used in more rigorous academic usability are very the similar to those used in academically oriented qualitative research otherwise. Although knowledge and comfort with the principles of usability research are important, internal team members capable of implementing a high-quality qualitative research protocol can adapt those tools and skills for usability evaluation. Additionally, more quantitative methods, such as user-reported usability scales or analytics (eg, click counts), collected on the back end of a software program, shed insight into how users interact with a tool [6,35-37].

The setting used for testing may be more elaborate for academic versus pragmatic usability testing; the former tends to reflect an emphasis on the representativeness of the testing environment, whereas the latter indicates the tendency to prioritize time and cost concerns over the achievement of a high-fidelity testing environment [36]. The number of participants also typically varies between academic and pragmatic usability with the recommendation for academic usability being a minimum of 15 participants, deemed representative of the intended end users, whereas in pragmatic usability testing, fewer participants may be considered sufficient to inform design decisions, particularly if testing is integrated into numerous rapid iterative and agile development and testing cycles [15,20]. Furthermore, academic usability studies may require enough subjects to be able to carry out meaningful statistical analysis or reach saturation of data, whereas this is typically not a requirement for pragmatic testing.

### Data Capture, Analysis, Reporting, and Dissemination

Although the methodologies employed may be similar across approaches, data capture and analysis is a key area of difference with the academic approach requiring more involved data capture to inform a level of analysis appropriate for an academic publication. Even though the pragmatic goals of a usability study can be met with detailed field notes, academic objectives may demand a full transcription of usability sessions reflecting a variety of types of data captured (eg, video, audio recording, screen captures, etc). Termination of data collection is based on the achievement of saturation for that iteration of the tool, as is common in traditional academic qualitative research, rather than on time and cost considerations [12,37].

Similarly, analytic methods differ across the 2 approaches. On one end of the spectrum, purely pragmatic projects might use only field notes, which may be loosely organized into practical usability themes and issues used in real time to inform build recommendations. On the other end of this spectrum is a heavily academic project with copious amounts of raw data to be analyzed systematically, as in a typical academic qualitative project; these data may even be combined with the analysis of more quantitative assessments for a mixed-methods approach to usability evaluation. Instant data analysis has emerged as a solution to reduce time and cost related to traditional (academic) usability evaluation while maintaining a systematic approach. However, while offering strategies for providing usability feedback to development teams efficiently, the data capture and analysis phase remain pragmatically rather than academically focused [12].

User feedback can be a useful marker indicating potential areas of focus for deeper learning during more rigorous qualitative analysis in the case of academically oriented studies. Although time-consuming, the depth and rigor of this type of data collection and analysis are necessary to uncover more subtle usability patterns and insights as well as produce high-quality findings fit for peer-review academic publication [38]. Given this, the depth of data capture and analysis as well as the format of reporting and dissemination are warranted. From the pragmatic perspective, summary reports highlighting usability issues and build recommendations suffice. Real-time summary documents can also be used to ensure the capture of key quotations from direct user feedback to be used to improve the tool at hand and drive changes in system design more broadly and therefore, they may be useful for academic objectives as well.

The choice of method and level of data analysis are the primary drivers of the difference in the time frame between academically versus pragmatically focused projects. An academically focused usability study may see value in conducting multiple rounds of various types of usability testing to achieve data saturation and analyzing audio, video, and screen capture data to uncover evidence to support findings relevant to the academic community. More pragmatic projects that incorporate usability testing may conduct just 1 cycle of 1 type of testing (eg, 1 cycle of think-aloud testing) with summary memos for prototype iteration but no further analysis of usability data [12,39].

### Hybrid Approach to Usability Testing

We believe the needs of both academic and pragmatic usability evaluation can be served by a hybrid approach. As described above, key drivers of differences in the features and cadence of academic versus pragmatic usability studies are the depth of data capture and analysis. With a hybrid approach, usability testing is tackled in the spirit of rapid, agile iteration while planning for the documentation needs required for deeper academically focused analysis. With attention paid to rigorous systematic data capture with a sufficient number of end users to meet academic objectives, in-depth qualitative or mixed-methods analysis can occur later in the product development lifecycle, although ideally before wide release of the optimized system, to ensure the opportunity for any later findings to find their way into final product iterations [21,38].

Teams best able to conduct this type of hybrid work are multidisciplinary and cross-functional, featuring some expertise in design thinking, agile product development, user interaction design, rapid pilot testing, and iteration in addition to team members with more traditional research HIT backgrounds [40]. While research and development teams conduct multiple usability testing cycles systematically, each session can be concisely summarized in a rapid fashion for tool iteration and to serve as a growing body of key feedback for the design team throughout the development process. This combined approach allows new development projects to efficiently iterate and optimize from usability data while preserving the potential for these projects to produce deeper insights via thorough qualitative analysis to inform further tool development and usability research by way of academically focused dissemination.

Our experience suggests that combining strategies for testing and evaluation provides a feasible approach equipped to meet academic objectives while also satisfying real-time needs of pragmatic usability evaluation. In this paper, we reviewed 2 case studies to demonstrate its feasibility and illustrate how this approach can be operationalized to build tools in a pragmatic, agile way while serving academic goals [32,41,42].

## Methods

Using a hybrid approach as a framework, we describe our experience incorporating usability evaluation in 2 HIT development projects [42-46]. These 2 case studies are used to illustrate the operationalization of a hybrid approach and demonstrate its potential value and feasibility. In the first case, we describe the adaptive design of an EHR CDS tool designed to reduce inappropriate antibiotic prescribing for upper respiratory infections. In the second case, we outline the design and development of a decision support tool-embedding goal setting into primary care EHR workflows. After a brief description of the project, we complete a side-by-side evaluation of each case study with regard to the key dimensions to consider in the design of a usability evaluation as outlined in Table 1.

This research did not involve human subjects. An institutional review board approval was not required because it did not involve a review of previously published data and did not involve data collection.

## Results

### Case Study 1: The Integrated Clinical Prediction Rule Decision-Support Tool

The objective of the Integrated Clinical Prediction Rule 2 (iCPR2) project, a National Institutes of Health (NIH)-funded research study, was to employ a user-centered approach to adaptively design an EHR CDS tool to reduce inappropriate antibiotic prescribing for upper respiratory infections and assess the adapted tool’s adoption and effectiveness [41,42]. By design, this project required relatively rapid incorporation of end user input and delivery of academic products related to lessons learned for the user-centered design of CDS tools.

The first phase of the study involved conducting laboratory-style usability testing of 12 clinician users who interacted with the guidelines embedded in the EHR by following a script driven by the experimenters. The participants were asked to verbalize their thoughts while interacting with the EHR and guidelines. While carrying out this study, technical staff was involved in implementing the guidelines observed the sessions. Based on their notes, they were immediately able to arrive at important modifications to the EHR and guidelines, satisfying pragmatic goals of the project. In addition, the study then moved to further phases in which more rigorous testing in near-live contexts was conducted prior to the actual release of the guidelines in the EHR for real use. This involved having users interact with a simulated digital patient to observe how the guidelines would be triggered in real-life contexts, followed by a formal clinical trial to assess the uptake of the guidelines. These latter objectives of the same study met the academic usability goals of providing publishable and useful knowledge that could guide further studies and other researchers in the future [31,32]. Thus, the approach could be considered to be hybrid in that it was designed to address both pragmatic short-term goals and objectives as well as longer-term scientific objectives for publication and knowledge dissemination.

### Case Study 2: The Avoiding Diabetes Thru Action Plan Targeting Tool

The Avoiding Diabetes Thru Action Plan Targeting (ADAPT) tool, also the product of an NIH-funded decision-support trial, was designed to support the integrated care counseling of prediabetes by providing templates within an EHR to guide physician-patient dialogues [44,45]. This study also involved conducting usability testing of clinician users as they interacted with the template embedded in the EHR, where they were asked to think aloud while interacting with the system and the templates. All the computer screens and audio were recorded and analyzed at the surface level for quick-fix problems and at a more detailed level of sufficient quality and reliability to lead to publishable journal results (to fulfill the goals of both pragmatic and academic usability engineering within the same study design).

With academic objectives in both cases, the decisions regarding methods used, setting, and the number of participants were made accordingly; data capture also reflected the downstream plan to transcribe and apply rigorous qualitative analysis; for example, in iCPR2, full-screen capture and audio were recorded for each think-aloud, near-live, and live usability session using Morae (think-aloud and near-live) and Camtasia (live) software. Researchers trained in usability methods also took detailed field notes [33]. The depth of data capture allowed researchers the ability to subsequently conduct a synchronous review of audio and video files together, allowing deeper analysis and results for the production of academically oriented findings suitable for dissemination in the scientific literature. Simultaneously, pragmatic objectives were recognized and addressed, as field notes were turned into summaries with recommendations to be considered for rapid tool modification.

In the case of ADAPT, pragmatically oriented summaries from usability session observations revealed that limited text length in the patient instruction field contributed to generic, nonpatient-specific content. A deeper qualitative analysis of the session data, including of the information entered in this field, further revealed that this content was unconducive to goal setting. Additionally, the in-depth analysis revealed a number of workflow issues, such as incompatibility of flow with encounters not focused on diabetes [44]. Both of these findings were important to the design of ADAPT but are also valuable for informing the design of other technologies with similar functionalities. Table 2 is a side-by-side comparison of the usability evaluation features of each of these two case studies.

**Table 2:** Case study comparison of usability evaluation features.

Feature and usability type		Case study 1 (Integrated Clinical Prediction Rule 2)	Case study 2 (Avoiding Diabetes Thru Action Plan Targeting)
**Objectives**			
	Academic	To generate evidence on the optimal adaptation of clinical decision-support tools	To generate evidence on the clinical impact of an electronic health record-enabled prediabetes counseling tool
	Pragmatic	Tool adaptation and identification of issues in tool build before widespread deployment	User feedback for recommendations to tool developers
Methods used: Academic and pragmatic		Direct observation Think-aloud Near-live Live testing Semistructured group interview (postdeployment)	Direct observation Think-aloud Near-live Live testing
Setting: Academic and pragmatic		Laboratory and *in situ*	Laboratory and *in situ*
Core team: Academic and pragmatic		9 members (expertise: primary care, clinical decision support, informatics, electronic health records, usability, qualitative research, and graphic design)	6 members (expertise: primary care, health psychology, diabetes education, nutrition, informatics, usability, and graphic design)
Number of participants: Academic and pragmatic		Think-aloud=12 clinicians Near-live=12 clinicians (same) Live=3 clinicians and 6 encounters Postdeployment=75 clinicians and 14 sites (group interviews)	Think-aloud=7 clinicians Near-live=6 clinicians
Data capture: Academic and pragmatic		Note taking Audio recording of sessions Video recordings Screen capture	Note taking Audio recording of sessions Screen capture
Termination criteria: Academic and pragmatic		Termination with data saturation for current iteration	Termination with data saturation for current iteration
**Data analysis**			
	Academic	Qualitative thematic analysis by 2 independent coders	Qualitative thematic analysis by 2 independent coders
	Pragmatic	Thematic analysis of observational field notes	Thematic analysis of observational field notes
**Output**			
	Academic	Detailed data tables and results reporting	Detailed data tables and results reporting
	Pragmatic	Summary reports from field notes	Summary reports from field notes
**Dissemination**			
	Academic	Publication of protocol and usability findings from think-aloud, near-live, and live testing in peer-reviewed journals	Publication of protocol and usability findings from think-aloud and near-live testing in peer-reviewed journals
	Pragmatic	Research team Electronic health record development team	Research team Electronic health record development team
**Time frame**			
	Academic	Think-aloud or near-live usability 16 months from the beginning of data capture to the publication of findings	Think-aloud or near-live usability 11 months from the beginning of data capture to the publication of findings
	Pragmatic	Think-aloud or near-live usability 2 months from the beginning of each phase of data capture to the completion of all summary reports	Think-aloud or near-live usability 1 months from the beginning of each phase of data capture to the completion of all summary reports

## Discussion

### Principal Findings

We reviewed 2 case studies to demonstrate the feasibility of a hybrid approach and illustrated how the approach could be operationalized to build tools in a pragmatic, agile way while serving academic usability research objectives. In both case studies, research teams were presented with pragmatic and academic objectives, necessitating the delineation of an approach to resolve what initially seemed to be a tension between the 2 approaches to usability research. While approaching the iCPR2 project, for example, with purely pragmatic usability methods, we would not have been able to produce and disseminate findings worthy of academic publication, missing the opportunity to enrich the body of evidence for the larger CDS and usability community. However, a purely academic approach to usability would have extended the development timeline of the iCPR2 tool, cutting into the time available to make and study tool iterations and the effects on process and clinical outcomes. When consideration of the needs of both perspectives are recognized and addressed, as in the hybrid approach, priorities can be negotiated upfront to produce a usability evaluation designed to produce a quality tool as well as usability findings of maximum value to the project team and the usability community at large.

As the need for rapid, user-centered HIT grows, efforts to develop effective technology tools to support evidence-based health care require an approach to systematic usability research that addresses both the pragmatic as well as academic needs of a project. At the crux of this hybrid approach is the collection of detailed audio and video data amenable to longer-term in-depth analysis, while rapidly collecting and summarizing information to drive system improvements in a short time frame (ie, within hours or days rather than weeks or months). The pragmatic, postsession summary memos and subsequent group solutioning supported agile development timelines, whereas the deeper qualitative analysis of the transcribed audio and video data generated more complex and orthogonal observations and insights for academic dissemination. Results from the in-depth qualitative analyses were applied prior to widespread system release in both projects but did not impede or preclude an agile development process or timeline.

This deeper analysis of data revealed additional important findings not apparent from the initial session summary memos obtained from observation as well as provided the data necessary for the rigorous analysis and reporting suited to addressing the project’s academic goals. This is evident in our publication of usability findings and implications from the ADAPT study in peer-reviewed publications [44-46]. Similarly, in the case of iCPR2, near-live session data captured workflow-sensitive usability problems missed in both the (pragmatic) field note summary document as well as in the think-aloud usability research cycle [33]. This finding indicates both the value of multiple rounds of usability testing with a variety of methods as well as the potential value added by the transcription and deeper analysis of session data. More complex analyses and insights, though more time-consuming to generate, have been valuable for optimizing our overall approach to developing similar CDS systems and thus provided generalizability of findings essential in academic research.

### Limitations

This evaluation of case studies prioritizes observational, qualitatively-focused methods over quantitative methodologies. This is not to negate the value of quantitative data sources to either academic or pragmatic usability research because a mixed-methods approach can be valuable to the objectives in both cases. Given the role that qualitative data capture and analysis play in the tension between academic and pragmatic usability evaluation, a focus on more qualitative usability research methods was deemed appropriate. This paper reports on 2 case studies in which the authors were leaders in the design and implementation, potentially limiting the generalizability of the finding that our approach is readily feasible for other teams in different contexts. Additionally, the data capture methods used were the same in both cases; analysis of cases with only a subset of data capture methods would offer additional insight into the application of the hybrid approach.

### Conclusions

We observed that the hybrid approach outlined in this paper was a feasible way to address the needs of academic usability and pragmatic usability objectives. Borrowing from industry usability testing practices common outside of academia and from our experience as illustrated by these 2 case studies, we have demonstrated that a hybrid approach can meet the needs of both by leveraging the rigor of academic usability testing along with the flexibility and rapid, agile characteristics of pragmatic usability methods. These studies provide novel examples of a hybrid approach that meets the needs of system developers charged with building and optimizing systems as well as academic usability researchers tasked with furthering our knowledge and perspective on the role of usability testing in health care technology.

## Abbreviations

ADAPT: Avoiding Diabetes Thru Action Plan Targeting
CDS: clinical decision support
EHR: electronic health record
HIT: Health Information Technology
iCPR2: Integrated Clinical Prediction Rule
NIH: National Institutes of Health
